# Medical and Philosophical News

**Published:** 1781-05

**Authors:** 


At a public meeting of the Academy of Surgery at Paris, on the 26th of April, the gold medal of 500 livres value was adjudged to the celebrated Professor Camper for the best dissertation on the prize question announced last year; after which were read the elogy of the late M. Disdier, by M. Louis; some reflexions on the exfoliation of bones by M. Fabre; remarks on abscesses in the substance of the tongue by M. Petibeau; and a dissertation on the locked jaw by M. Sabatier.

